# Benefits, risks, and cost-effectiveness of COVID-19 self-tests from a consumer’s perspective

**DOI:** 10.1186/s12913-021-07277-4

**Published:** 2022-01-10

**Authors:** Afschin Gandjour

**Affiliations:** Frankfurt School of Finance & Management, Adickesallee 32-34, 60322 Frankfurt am Main, Germany

**Keywords:** COVID-19, Self-test, Cost-effectiveness

## Abstract

**Background:**

The aim of this study is to quantify the health benefits, risks, and cost-effectiveness of COVID-19 self-tests from a consumer’s perspective in Germany.

**Methods:**

The analysis is based on a modelling approach using secondary data. The clinical endpoints considered in this analysis are avoided SARS-CoV-2 infections and secondary severe clinical events (death, intensive care unit (ICU) admission, and long COVID syndrome). The study determines the number of self-tests that need to be conducted under a 7-day incidence of 75 per 100,000 population to prevent one infection or severe clinical event. Furthermore, the study calculates the cost of testing per avoided clinical event and quality-adjusted life year (QALY) gained from a consumer perspective.

**Results:**

Disregarding the rate of unreported COVID-19 cases, 4556 self-tests need to be conducted (over 12 years) in order to avoid one undesirable event (death, intensive care unit stay, or long COVID syndrome). Ninety percent of infections are not avoided among direct contacts but along the chain of infection. The costs per quality-adjusted life year gained from a consumer’s perspective are €5870. This ratio is particularly sensitive to the 7-day incidence, effective reproduction number, and the age of contacts.

**Conclusions:**

The benefits of self-testing in the general population at a 7-day incidence rate of 75 per 100,000 appear to be minor. Nevertheless, cost-effectiveness may still be acceptable in the presence of higher-risk contacts given the low costs of self-test kits in Germany.

## Introduction

Since March 6, 2021, the German discounter Aldi has been offering COVID-19 antigen rapid tests (self-test kits) for lay use/at-home testing. Other supermarket chains and drugstores have followed suit. Home collection and at-home tests that are available without a prescription “may be called “direct-to-consumer” tests [1]. In Germany, self-tests must be approved by the German Federal Institute for Vaccines and Biomedicines (Paul Ehrlich Institute) and have a sensitivity of > 80% in symptomatic patients within 7 days of the onset of symptoms and a specificity of > 97% in asymptomatic persons with no “concrete” exposure risk [2]. While evidence suggests that sensitivity in asymptomatic patients may be less than 80%, in patients with a high viral load it may be above 80% [3, 4]. Prices of self-tests are relatively cheap in Germany (less than €1 per piece), while in countries such as the United States even lowest prices are approximately 10-fold higher [5].

In Germany, certain public spaces are currently only accessible to those who are vaccinated, recovered, or tested (the “3G” rule). In principle, self-tests are also permitted on site under supervision. This means that a test can also be taken under the supervision of the person responsible for compliance with the 3G rule, e.g., in a hairdressing salon.

In Germany, the current dominant strain of SARS-COV-2 is the Delta variant. The current full vaccination rate is 72% between the ages 18 and 59 years and it is 84% at or above the age of 60 years [6].

The aim of this study is to quantify the benefits, risks, and cost-effectiveness of COVID-19 self-tests from the perspective of a German consumer. Consumers and their contacts were assumed to be representative of the German population. In other words, they were assumed to be at an average risk for infection with the Delta variant and to have an average disease course if infected. A consumer perspective is adopted because the costs of self-tests are entirely borne by the consumer. A consumer perspective is compatible with a cost-effectiveness analysis [7].

## Methods

### Conceptual approach

This analysis is based on an analytical model (i.e., a mathematical model with a closed form solution) that can capture the binary outcomes of interest as described below. The model uses secondary data. All methods were carried out in accordance with the relevant guidelines and regulations.

In the base case, I assumed contacts at average risk of infection with the Delta variant and an average disease course in the event of an infection. The probability of transmission and outcomes of COVID-19 consider the proportion of the population that is fully vaccinated and the vaccine effectiveness. The likelihood of infections in contacts also depends on the regional SARS-CoV-2 incidence including the number of unreported cases, the viral strain underlying the infection, the number of contacts, and general COVID-19 containment measures. The harm of an infection depends on the risk factors of contacts (e.g., age).

The benefits of self-tests are measured by the number of SARS-CoV-2 infections avoided in contacts. In addition, the study goes beyond determining contagiousness by capturing severe clinical events following an infection (death, intensive care unit (ICU) admission, and long COVID syndrome). To avoid double counting of severe clinical events, only non-fatal ICU stays were taken into account. A combined endpoint consisting of death, ICU admission, and long COVID syndrome was defined, and the components were weighted according to their incidence.

The study does not take non-severe symptoms of COVID-19 into consideration assuming that testing is not motivated by the desire to avoid non-severe symptoms in contacts (otherwise influenza testing for the purpose of avoiding non-severe infections would be much more prevalent). In addition, the study computes the cost of testing per avoided event and per quality-adjusted life year (QALY) gained. The time horizon was set to less than 1 year, which is sufficiently long to capture all clinical events (especially long COVID syndrome) and transmissions following the index case infection.

The comparator of self-testing is the next effective intervention, which is the absence of self-testing but the maintenance of personal protective measures. In the counterfactual scenario without self-testing, infected contacts infect their contacts and so on, resulting in a chain of infection that spreads in this manner. To calculate the number of infections avoided by self-tests over the entire chain of infection, the convergence of a geometric series with quotient *q* of two adjacent elements of the sequence is considered: $\sum_{k=0}^{\infty }{q}^k=\frac{1}{1-q}$. In this analysis, variable *q* corresponds to the effective reproduction number *R*, i.e., the number of people infected by the index case.

In a sensitivity analysis, an additional lowering of the R value caused by an increase in utilization of rapid tests in the population was calculated using a mathematical formula. According to equation 13 in Kuniya and Inaba [8], there is a linear relationship between the R value and the reciprocal of the product of the population test rate and the sensitivity. Given that the testing rate does not equal the testing frequency, a logarithmic relationship of the following form was assumed: testing rate = −ln(1 - testing frequency) [9].

By multiplying the 7-day incidence per 100,000 population, test sensitivity, and R value and dividing the product by the number of infections avoided over the entire infection chain, the number of self-tests that need to be conducted to prevent exactly one infection is calculated. By dividing the number by the probability of a combined endpoint, the number of self-tests that need to be conducted to prevent one severe clinical event is determined.

The advantages of self-testing are traded off against the disadvantages, i.e., false negative and false positive results. To determine false positive results, the number of tests required to prevent one severe event was multiplied by 1 minus specificity. Furthermore, the case is considered in which the consumer neglects personal protective measures such as the so-called AHA + L rule (social distancing, hand hygiene, wearing face masks, and ventilation) after a negative self-test. In this case of a false negative test result the number of infections and clinical events increases. The increase in infections is calculated by multiplying the 7-day incidence, 1 minus test sensitivity, the increase in the effective R value without protective measures (considering its linear dependence on the fraction of immunized individuals [10]), and the number of infections avoided along the entire chain of infection.

Moreover, from a consumer perspective, the cost of testing per avoided clinical event and per QALY gained is determined. The gain in QALYs reflects the health gain from avoiding death, ICU admission, and long COVID syndrome. To compute the number of QALYs from preventing death, the analysis multiplied the remaining life expectancy at the average age of death from COVID-19 with the preference weight over the same period. To calculate QALYs gained from avoiding ICU admission, the remaining life expectancy of ICU patients was deducted from the remaining life expectancy in the general population. In addition, the loss of quality of life in ICU patients was considered.

Furthermore, I analyzed the relationship of testing years to avoid one clinical event and costs per QALY gained with respect to the age of contacts. To this end, I applied age-specific infection fatality rates (IFRs) and probabilities of ICU admission. To determine probabilities of ICU admission, I applied the age-gradient of the IFR assuming that ICU case fatality is independent of age (as a younger age of ICU patients has not been associated with a change in ICU fatality [11]). For the age group 80+ years this approach leads an overestimation of the ICU admission rate, however, because of the significant share of deaths occurring in nursing homes (approximately 25% [12]). For this age group I therefore applied the age-specific hospital admission rate as a proxy. Of note, for age groups below 80 years, data on hospital admission rates [13] did not match the age groups for IFR and hence was not used.

The base-case analysis does not consider downstream costs associated with clinical events and the costs of false positive tests because they are largely borne by social health insurance and employers. In a sensitivity analysis, the maximum co-payment, which is 2% of the gross household income [14], was applied.

### Sensitivity analysis

One-way deterministic analyses assessed parameter uncertainty by varying the input parameters that were subject to variation one at a time.

### Data

#### Clinical and epidemiological data

Table 1 presents all input values and distributions used in the base case and sensitivity analysis. In the base case, a 7-day incidence of 74.7 cases per 100,000 population (as of September 17, 2021 [15]) was applied. In a sensitivity analysis, the number of unreported cases, which is currently estimated to be twice the number of reported cases based on a systematic review of seroprevalence studies in Germany [16], was considered.

**Table 1:** Input values and distributions used in the base case and sensitivity analysis

Input	Mean (range)	Reference
*Epidemiological and clinical data*		
IFR in Germany (without vaccine)	0.014 (0.011–0.017)	[17, 18]
IFR by age (without vaccine)	Age-specific	[17–19]
Percent fully vaccinated		[6]
Age 18–59	0.72	
Age 60+	0.84	
Vaccine effectiveness against death		[20]
Age 18–59	0.96	
Age 60+	0.90	
Vaccine effectiveness against ICU admission, age 18+	0.93	[20]
Lost life years due to COVID-19	6.5	[21]
Preference weight in the absence of COVID-19	0.8	[22]
Probability of ICU admission	0.026	[19]
ICU mortality rate	0.26	[19]
Preference weight after ICU discharge	0.58	[23]
Probability of hospital admission, age 80+	0.29	[13]
Life expectancy after ICU discharge, years	2.1	[24, 25]
Prevalence of long COVID syndrome	0.025 (0.025–0.117)	[26]
Preference weight of long COVID syndrome	0.53 (0.42–0.64)	[27]
7-day incidence rate	65 (30–200)	
Proportion of undetected COVID-19 cases	0.50	[16]
Asymptomatic infection rate	0.191	[28]
Symptomatic infection rate	0.254	[28]
Average incubation period, days	5.8	[29]
Average infectious period, days	14	[30]
*Test data*		
Test sensitivity	0.81 (0.70–0.90)	[2]
Test specificity	0.98	[2]
Rapid COVID-19 antigen self-test, €	2 (0.80–2)	
*Additional data*		
Annual gross household income, €	56,808	[31]

*ICU* intensive care unit, *QALY* quality-adjusted life year

IFRs and ICU admission rates were adjusted for the proportion of individuals with full vaccination as well as the vaccine effectiveness against death and ICU admission. Vaccine effectiveness was based on real-world data from Germany collected during calendar weeks 37 to 40 [6].

In a UK study, the probability of a long COVID syndrome at 12 weeks was 3% based on continuous symptoms [26]. That is, any of 12 symptoms were reported at consecutive visits at an approximately monthly interval [26]. In a matched control group, the probability of symptoms at 12 weeks was 0.5%. I determined the weekly excess probability over the available time horizon of 18 weeks, effectively conducting an area under the curve analysis. In a sensitivity analysis, I used the percentage of self-reported long COVID syndrome (11.7%) [26].

The effective R value with the current test strategy (i.e., approximately 1 million PCR tests per week [32] and almost 500,000 rapid tests (excluding self-tests) per day [33]) was 0.90 on September 17, 2021 [34]. In other words, a COVID-19 patient infected 0.90 other people on average.

In a sensitivity analysis, the reduction in the R value due to increased utilization of rapid tests in the population was determined. Based on 1 billion self-tests and rapid tests that the federal government has secured for 2021 [35], around 4% of the population could be tested daily or 28% of the population could be tested weekly between March and December 2021. To calculate the reduction in the R value, the incubation time and transmission rate of symptomatic and asymptomatic COVID-19 cases caused by the Delta variant were considered.

The minimum levels of the Paul Ehrlich Institute [2] for the sensitivity and specificity of self-tests (81 and 98%, respectively) were used.

#### Cost-effectiveness data

The median age of death of COVID-19 patients until June 22, 2021, was 84 years, resulting in an estimated loss of 6.5 life years [21]. The preference weight was based on the EQ-5D-5L index score in the German general population at or above the age of 75 years [22].

For ICU survivors a preference weight (EQ-5D-3L index score) based on a sample of UK patients with acute respiratory distress syndrome surveyed 12 months after ICU discharge [23] was applied.

For patients with long COVID syndrome, I used a preference weight for patients with chronic fatigue syndrome, which shows many overlaps with the long COVID syndrome [36]. To this end, I determined the average of the minimum and maximum preference weight on adult patients (based on the EQ-5D) that were reported in a systematic review on the cost-effectiveness of interventions on the chronic fatigue syndrome [27]. The range of values was tested in a sensitivity analysis.

## Results

### Base case

Based on the current 7-day incidence, test sensitivity and R value, approximately 1836 (= 75/100,000 ⋅ 0.81 ⋅ 0.90) tests need to be conducted to prevent an infection in a direct contact. However, the impact of the otherwise infected contact on their contacts is not yet taken into account here. If the otherwise untested index case infected 0.90 of their contacts and the latter in turn infected 0.90 of their contacts and so on, an infection chain spreading by a factor of 0.90 would result in 10 infections.

Consequently, approximately 184 self-tests need to be conducted to avoid one infection. This corresponds to approximately 39,510 self-tests to prevent one death, two ICU admissions, and six long COVID cases (i.e., 4556 self-tests to prevent one undesirable event). On the other hand, 4556 self-tests with a specificity of 98% require approximately 91 PCR tests or isolations, which can be traced back to false positive results. In other words, a consumer would have to test herself daily for 12.5 years to prevent one undesirable event (death, ICU admission, or long COVID syndrome). The consumer would have to isolate herself for 1 day almost every alternate month. The costs per avoided event are €9111, whereas the costs per QALY gained are €5870.

**Fig. 1:**
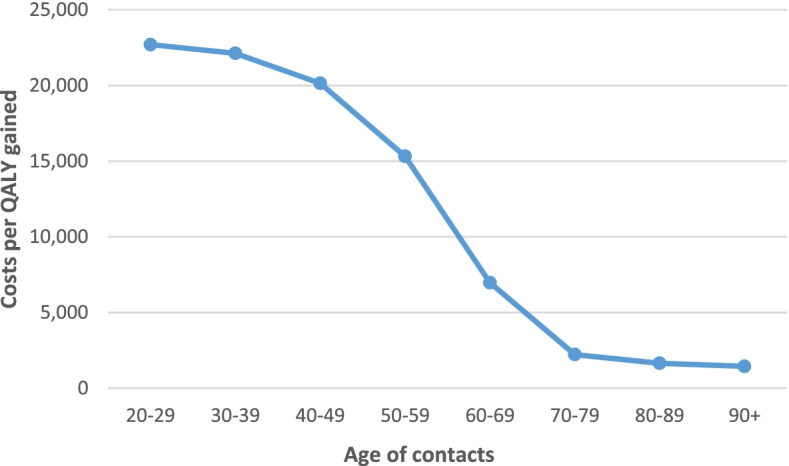
Costs per quality-adjusted life year gained (QALY) depending on the age of contacts

**Fig. 2:**
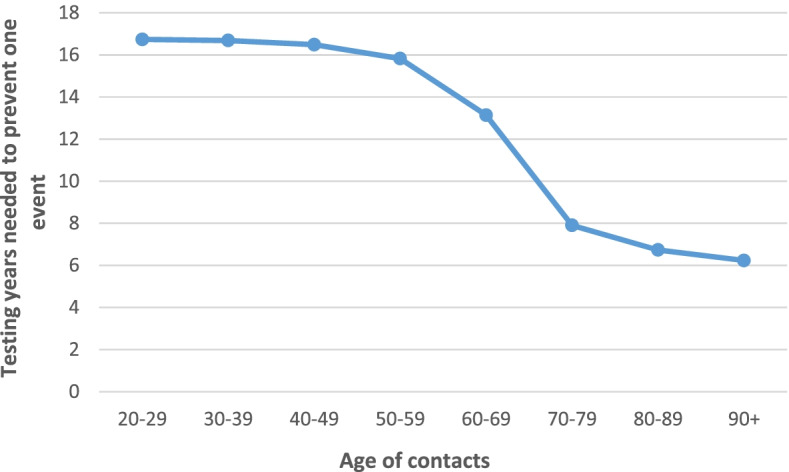
Testing years to avoid one clinical event depending on the age of contacts

Furthermore, as shown in Figs. 1 and 2, the age gradient of contacts both for testing years to avoid one clinical event and costs per QALY gained is steep (even more pronounced for the latter endpoint). This reflects the age gradient of the IFR of COVID-19.

### Sensitivity analysis

**Fig. 3:**
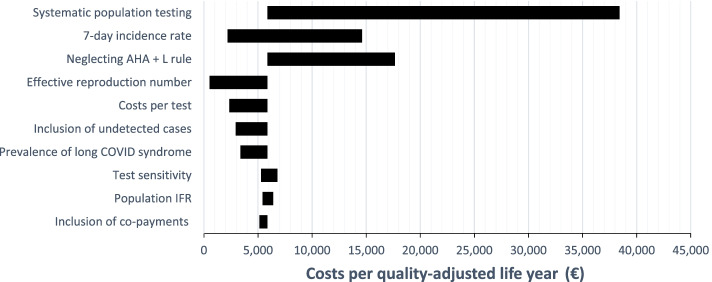
Tornado diagram demonstrating the results of the one-way sensitivity analysis. The variables are ordered by the impact on costs per quality-adjusted life year gained

Results of the one-way sensitivity analysis are displayed in the tornado diagram in Fig. 3. The input with the greatest impact on the cost-effectiveness ratio is mass testing in the population due to its impact on the R value. If an additional 4% of the population is tested daily with a sensitivity of 81%, the R value drops to 0.58, resulting in a considerable reduction in the benefits of self-testing and an increase in the cost-effectiveness ratio. In addition, the cost-effectiveness ratio is particularly sensitive to the 7-day incidence rate, with a higher incidence leading to improved cost-effectiveness.

If undetected COVID-19 cases are included, the 7-day incidence is two times higher. Accordingly, a consumer would have to test herself daily for about six years to prevent an undesirable event (death ICU admission, or long COVID syndrome).

If personal protective measures against SARS-CoV-2 infection are neglected after a negative self-test, the benefit of self-testing is reduced to one-third. Thus, a consumer would have to test herself daily for about 38 years to prevent an undesirable event (death, ICU admission, or long COVID syndrome).

## Discussion

As shown in this study, the cost-per-QALY ratio of self-tests depends particularly on the R value, the 7-day incidence, and the age of contacts. The number of self-tests/testing years to prevent one undesirable event (death, ICU admissions and long COVID syndrome) exhibits similar patterns. Taking the number of unreported cases into consideration reduces the number of tests to prevent an undesirable event by 50%, while neglecting the AHA + L rule in private surroundings results in a threefold increase. While such a change in behavior does not completely negate the benefits of self-tests, an individual of the average age in the German population (45 years) would barely be able to enjoy the benefits within her remaining lifetime at the current level of incidence.

As a general rule, the greatest advantage of self-testing is not the avoidance of infection in direct contacts, but the positive external effect (91% of the infections avoided). The benefit of rapid tests decreases with the increase in the number of citizens tested (as this decreases the R value).

While the benefits of testing appear to be minor, the costs of testing appear to be minor as well, resulting in perhaps still acceptable cost-per-QALY ratios in the presence of higher-risk contacts. Nevertheless, cost-per-QALY ratios should be interpreted with caution because they cannot be directly compared against cost-effectiveness thresholds that may be used from a social health insurance or societal perspective. Instead, cost-per-QALY ratios reflect private willingness to pay.

Perhaps counterintuitively, self-test producers may be justified in commanding higher test prices when incidence rates increase because of larger testing value. Nevertheless, higher prices may reduce demand, thus entailing less control of the pandemic and a prolongation of the period of voluntary and/or lockdown restrictions.

The reduction in the R value by 0.32 calculated according to the formula by Kuniya and Inaba [8] does not present an overestimate, at least if the results by Gorji et al. [37] are considered. According to the latter, weekly testing of 25% of the population with a sensitivity of 85% even leads to a reduction in the R value of about 0.35 (Fig. S2 in Gorji et al. [37]). However, the analysis does not take into account whether a small population is tested frequently or a larger population is tested less frequently. According to P. Jenny (personal communication, March 12, 2021), the latter leads to a greater reduction in the R value.

As a limitation, the analysis does not take into account the feeling of security when meeting other individuals after a negative test. As a further limitation, the loss in life years due to death from COVID-19 is overestimated because COVID-19 patients have more comorbidities than individuals in the same age cohort of the general population. In addition, their time preference is not considered. On the other hand, the loss in life years is underestimated because death from COVID-19 in younger age groups entails a relatively high loss of life years that does not decrease linearly with age.

In terms of the transferability and relevance of the results and conclusions of this study to other countries, the usual caveats apply. The specific reasons for caution include between-country differences in clinical and epidemiological data, testing costs, and the consumers’ willingness to pay for health benefits.

In summary, this analysis provides information about the benefits of self-tests based on previously disregarded endpoints. The benefits of self-testing in the general population at a 7-day incidence rate (75 per 100,000) appear to be minor while cost-effectiveness may still be acceptable with higher-risk contacts. Improvements in test accuracy due to technological advancements would require a recalculation.

## Data Availability

All data are contained within the manuscript. The data sources are listed in Table 1 and are publicly available.

## Abbreviations

AHA + L: Social distancing, hand hygiene, wearing face masks, and ventilation
COVID-19: Coronavirus disease 2019
EQ-5D-5L: EuroQoL-5-dimensions-5-level
ICU: Intensive care unit
IFR: Infection fatality rate
QALY: Quality-adjusted life year
PCR: Polymerase chain reaction
R: Effective reproduction number
SARS-CoV-2: Severe acute respiratory syndrome coronavirus 2
